# Glucose Intolerance on Phaeochromocytoma and Paraganglioma—The Current Understanding and Clinical Perspectives

**DOI:** 10.3389/fendo.2020.593780

**Published:** 2020-11-26

**Authors:** Ichiro Abe, Farhadul Islam, Alfred King-Yin Lam

**Affiliations:** ^1^ Cancer Molecular Pathology of School of Medicine, Griffith University, Gold Coast, QLD, Australia; ^2^ Department of Endocrinology and Diabetes Mellitus, Fukuoka University Chikushi Hospital, Chikushino, Fukuoka, Japan; ^3^ Department of Biochemistry and Molecular Biology, University of Rajshahi, Rajshahi, Bangladesh

**Keywords:** phaeochromocytoma, glucose intolerance, insulin secretion, insulin resistance, paraganglioma

## Abstract

Half of the patients with phaeochromocytoma have glucose intolerance which could be life-threatening as well as causing postoperative hypoglycemia. Glucose intolerance is due to impaired insulin secretion and/or increased insulin resistance. Impaired insulin secretion is caused by stimulating adrenergic α2 receptors of pancreatic β-cells and increased insulin resistance is caused by stimulating adrenergic α1 and β3 receptors in adipocytes, α1 and β2 receptors of pancreatic α-cells and skeletal muscle. Furthermore, different affinities to respective adrenergic receptors exist between epinephrine and norepinephrine. Clinical studies revealed patients with phaeochromocytoma had impaired insulin secretion as well as increased insulin resistance. Furthermore, excess of epinephrine could affect glucose intolerance mainly by impaired insulin secretion and excess of norepinephrine could affect glucose intolerance mainly by increased insulin resistance. Glucose intolerance on paraganglioma could be caused by increased insulin resistance mainly considering paraganglioma produces more norepinephrine than epinephrine. To conclude, the difference of actions between excess of epinephrine and norepinephrine could lead to improve understanding and management of glucose intolerance on phaeochromocytoma.

## Introduction

Phaeochromocytoma is a neuroendocrine tumor derived from chromaffin cells in the adrenal medulla that produces catecholamines (1, 2). There are recent advances on molecular pathogenesis as well as on the clinical perspectives in the tumor (1, 3). Common clinical complications in patients with phaeochromocytoma include hypertension, glucose intolerance, and cardiovascular dysfunctions (4). Among these complications, glucose intolerance attracts attention as recent developments noted regarding the mechanisms. Commonly, glucose intolerance is known for its hyperglycemia due to impaired glucose homeostasis, which is a result of impaired insulin secretion and/or increased insulin resistance (5, 6). In patients with phaeochromocytoma, Wilber and co-workers reported that insulin secretion was inhibited by an excess of catecholamine (7). In addition, Deibert and co-workers showed that insulin resistance was increased by excess catecholamine (8). Thus, impaired insulin secretion as well as increased insulin resistance could affect glucose intolerance in patients with phaeochromocytoma. Recent studies started to elucidate the mechanisms of glucose intolerance in phaeochromocytoma. In this review, we aim to summarize these recent findings of glucose intolerance in patients with phaeochromocytoma.

## Glucose Intolerance On Phaeochromocytoma: Incidents and Issues

Glucose intolerance is one of the complications of phaeochromocytoma and often found in patients with phaeochromocytoma (9, 10). Elenkova and co-workers revealed that half of the patients (93/186) with phaeochromocytoma had glucose intolerance (11). Beninato and co-workers also investigated 153 patients with phaeochromocytoma, showing that 23% (n=36) had diabetes mellitus. Of these, and 79% (n=33) had complete resolution of diabetes mellitus after extirpation of phaeochromocytoma (12).

Elenkova and co-workers indicated that patients with glucose intolerance had increased urine metanephrine (metadrenaline; metabolite of epinephrine/adrenaline) and normetanephrine (a metabolite of norepinephrine/noradrenaline) and were older than those without glucose intolerance (11). There were no significant differences among the two groups with respect to tumor size and body mass index. On the other hand, Beninato and co-workers indicated patients with diabetes mellitus had larger tumors than those without diabetes mellitus but there was no significant difference between the two groups in patients’ age and catecholamine values (12). Considering the different results of two studies, it remains controversial about the relationship between the incidence of glucose intolerance and the other clinical parameters. Thus, it is difficult to predict the risk of glucose intolerance in patients with phaeochromocytoma.

Almost all of the patients treated as diabetes mellitus before diagnosed phaeochromocytoma were initially diagnosed as type 2 diabetes mellitus. Glucose intolerance in patients with phaeochromocytoma is often difficult to be well-controlled and insulin therapy is sometimes needed. Nevertheless, a few cases were diagnosed as type 1 diabetes mellitus because of their impaired insulin secretion and necessity of insulin therapy (13, 14). In addition, the clinical manifestations of glucose intolerance in patients with phaeochromocytoma are sometimes severe. There were some cases of diabetic ketoacidosis or hyperglycemic hyperosmolar syndrome, which could be lethal without accurate therapy in patients with phaeochromocytoma (15–17). These previous reports indicated glucose intolerance in patients with phaeochromocytoma could exhibit severe phenotypes and must not be overlooked.

Glucose intolerance of phaeochromocytoma is a problem in the perioperative period. After tumor extirpation, hypoglycemia occurs to some patients with phaeochromocytoma. Akiba and co-workers demonstrated that 13% of patients had severe hypoglycemia after extirpation of phaeochromocytoma. This postoperative hypoglycemia is likely due to a sharp decrease of catecholamines by tumor extirpation (18). Recently, Araki and co-workers showed patients who had higher epinephrine and those who had glucose intolerance preoperatively were more likely to develop postoperative hypoglycemia, which indicated patients with higher epinephrine and glucose intolerance must be careful observation in the perioperative period (19).

## The Molecular Adrenergic Mechanisms Related to Glucose Intolerance and The Differences Among Types of Catecholamine Excess In Glucose Intolerance

Multifarious adrenergic receptor (α1, α2, β1, β2, and β3) and their different functions were noted in various organs (20). In patients with phaeochromocytoma, various adrenergic receptors were reported to be responsible for the glucose intolerance (21–26). Impaired secretion of insulin by catecholamines on phaeochromocytoma is caused mainly through adrenergic α2 receptors of β-cells in pancreatic islets (21, 22). The α2 receptors are divided into 3 subtypes (α2A, α2B, and α2C). Among them, α2A receptors were reported to suppress insulin secretion from pancreatic β-cells. Fagerholm and co-workers showed that adrenergic α2A receptor knockout mice or adrenergic α2 receptor antagonists exhibit elevated insulin levels and reduced glucose levels (21).

On the other hand, increased insulin resistance on phaeochromocytoma is caused by stimulated gluconeogenesis and glycogenolysis in the liver, which arises from an excess of glucagon and increase of free fatty acids as well as elevated glucose uptake in skeletal muscle. Stimulation of adrenergic β2 and α1 receptors of the pancreatic α-cells causes an increase in glucagon secretion (21, 23–25). Two previous *in vitro* studies demonstrated stimulation of adrenergic β receptors could increase glucagon secretion through elevated intracellular cAMP and Ca^2+^ signaling (23, 24). Among the β receptors, Philipson showed adrenergic β2 receptors could have most effects on glucagon secretion among adrenergic β receptors (25). Vieira and co-workers showed adrenergic α1 receptors antagonists stimulated glucagon secretion (23). In addition, they also showed adrenergic α2 receptor antagonists and agonists did not affect glucagon secretion although it was reported adrenergic α2 receptors were detected in pancreatic α-cells (21, 23).

In adipocytes, stimulation of the adrenergic β3 and α1 receptors affects the production of fatty acids (26, 27). de Souza and co-workers showed stimulation of adrenergic β3 receptors increased intracellular cAMP and elevated lipolysis, which caused increased fatty acid (26). In addition, Boschmann and co-workers showed that stimulation of adrenergic α1 receptors with adrenergic α1 receptor agonists led to promoting lipolysis and results in increased fatty acid (27).

Regarding glucose uptake in skeletal muscle, Shi and co-workers showed stimulating adrenergic α1 receptors increased glucose uptake in skeletal muscle with elevated leptin signaling with adrenergic α1 receptors deficient and transgenic mice (28). Shiuchi and co-workers also showed that adrenergic β2 receptors in skeletal muscle could mediate induction of glucose uptake in skeletal muscle by leptin signaling investigating adrenergic β receptor-deficient mice and forced expression of adrenergic β2 receptors (29). In addition, Mukaida and co-workers showed stimulating adrenergic β2 receptors which promoted glucose transporter type 4 (GLUT4) translocation (a protein acts as glucose transporter found in adipose tissue and striated muscles) and glucose uptake in skeletal muscle using adrenergic β3 receptor-deficient mice with adrenergic β2/β3 receptor agonists (30).

Epinephrine and norepinephrine were reported to have different affinities to respective adrenergic receptors. Epinephrine was reported to have a higher affinity to adrenergic α2 and β2 receptors than norepinephrine. On the contrary, norepinephrine was reported to have a higher affinity to adrenergic α1 and β1 receptors than epinephrine. Moreover, epinephrine and norepinephrine were reported to have a similar affinity to adrenergic β3 receptors (31).

**Figure 1:**
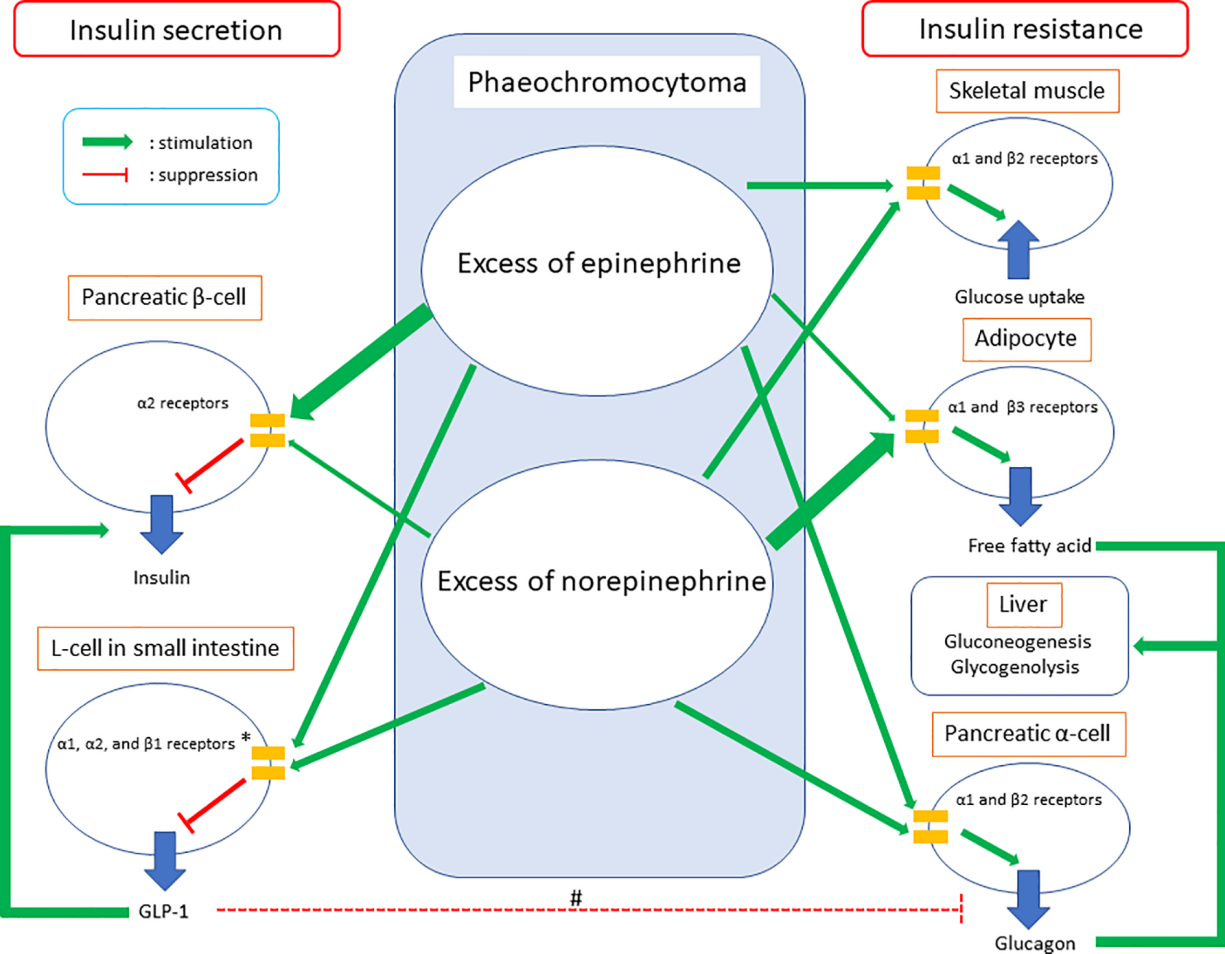
Summary of glucose intolerance on phaeochromocytoma. GLP-1, glucagon-like peptide-1. *The clinical study demonstrated GLP-1 secretion could be suppressed on phaeochromocytoma. Meanwhile, *in vivo* and *in vitro* studies showed epinephrine could increase GLP-1 secretion through adrenergic α1, α2, and β1 receptors and norepinephrine could inhibit GLP-1 secretion through adrenergic α1 and/or α2 receptors. #GLP-1 inhibits glucagon secretion in pancreatic α-cells commonly, while glucagon secretion was suppressed in the clinical study which investigated GLP-1 secretion on phaeochromocytoma. Considering both epinephrine and norepinephrine could increase glucagon secretion in pancreatic α-cells directly together, the study did not clarify whether increased GLP-1 secretion on phaeochromocytoma could inhibit glucagon secretion in pancreatic α-cells or not.

These molecular findings indicated an excess of catecholamine in phaeochromocytoma could lead to both impaired insulin secretion and increased insulin resistance through various adrenergic receptors as well as different actions between epinephrine and norepinephrine in them (**Table 1**, **Figure 1**).

**Table 1:** Different effects on glucose intolerance among adrenergic receptors.

Adrenergic receptors	Mechanism of effect on glucose intolerance	Affinity of epinephrine and norepinephrine
α1 receptors	fatty free acid ↑, glucagon secretion↑, glucose uptake in skeletal muscle↑, GLP-1 secretion ↓	Norepinephrine > Epinephrine
α2 receptors	insulin secretion↓, GLP-1 secretion ↓	Epinephrine > Norepinephrine
β1 receptors	GLP-1 secretion↑	Norepinephrine > Epinephrine
β2 receptors	glucagon secretion↑, glucose uptake in skeletal muscle↑	Epinephrine > Norepinephrine
β3 receptors	fatty free acid ↑	Epinephrine = Norepinephrine

GLP-1, glucagon-like peptide-1.

## Clinical Studies: Impaired Insulin Secretion and Increased Insulin Resistance in Patients With Phaeochromocytoma

Clinical studies revealed patients with phaeochromocytoma had impaired insulin secretion (32–34). Komada and co-workers demonstrated impairment of insulin secretion, particularly in an early phase of the insulin secretory response in 13 patients with phaeochromocytoma/paraganglioma (extra-adrenal phaeochromocytoma) (11 with phaeochromocytoma and two with paraganglioma) (32). They noted that improved insulin secretion after surgical removal of phaeochromocytoma/paraganglioma with hyperglycemic clamps as well as an oral glucose tolerance test. Moreover, the study investigated insulin resistance with hyperinsulinemic-euglycemic clamps and homeostasis model assessment of insulin resistance (HOMA-IR), which is surrogated marker of insulin resistance. There were no significant changes of insulin sensitivity index on hyperinsulinemic-euglycemic clamps from pre-operation to post-operation although postoperative HOMA-IR value was significantly improved than preoperative value, which indicated increased insulin resistance in patients with phaeochromocytoma was controversial in the study (32).

Petrák and co-workers demonstrated impaired insulin secretion and glucose intolerance in patients with pheochromocytoma using meal test and the homeostasis model assessment of β-cell function (HOMA-β), which is surrogated marker of insulin secretion. In the study, they indicated that impaired insulin secretion could be due to impaired glucagon-like peptide 1 (GLP-1) secretion (33) (**Figure 1**). Meanwhile, the study revealed significant increment of glucagon value and no changes in insulin/glucagon ratio between the preoperative and postoperative period. Commonly, GLP-1 decreases glucagon secretion (34). Considering excess of catecholamine leads to elevation of glucagon through adrenergic β2 and α1 receptors together, the results of the study about glucagon secretion might indicate there exists some unknown mechanism of glucagon secretion in patients with phaeochromocytoma (**Figure 1**).

There were clinical studies which revealed insulin resistance improved after adrenalectomy in patients with phaeochromocytoma as well as those related to the improvement of insulin secretion (35–38). Blüher and co-workers demonstrated increased insulin resistance in three patients with phaeochromocytoma having hyperglycemia medicated with anti-diabetic agents, which improved after surgical removal of phaeochromocytoma (35). A follow-up study by the same group revealed an excess of catecholamine-induced glucose intolerance through increased insulin resistance with the hyperinsulinemic-euglycemic clamp study in 10 patients with phaeochromocytoma (36). Moreover, Diamanti-Kandarakis and co-workers investigated glucose intolerance in 5 patients with phaeochromocytoma using oral glucose tolerance test and hyperinsulinemic-euglycemic clamp. The results demonstrated not only the improvement of glucose intolerance but also reduced insulin resistance by tumor extirpation (37). Recently, Guclu and co-workers investigated 44 patients with phaeochromocytoma using HOMA-IR and noted 65.9% of them (29 patients) had increased insulin resistance (38). Considering the results of these studies, both impaired insulin secretion and increased insulin resistance could induce glucose intolerance in patients with phaeochromocytoma. However, it has remained unclear whether the main factor of glucose intolerance is impaired insulin secretion or increased insulin resistance.

Glucose intolerance in patients with phaeochromocytoma could be improved by tumor extirpation. Meanwhile, there could be cases who could not receive tumor removal because of their complications or having metastatic phenotypes. Diamanti-Kandarakis and co-workers showed administrations of non-selective adrenergic α blocker and β blockers could improve glucose intolerance in patients with phaeochromocytoma, while the effects of the agents were significantly lower than those of tumor extirpation (37). This study indicated glucose intolerance in patients who could not undergo tumor removal could be difficult only by agents of adrenergic receptor antagonists.

## The New Insight: Differences in The Actions On Glucose Intolerance Among Types of The Excess of Catecholamine On Phaeochromocytoma

DiSalvo and co-workers showed epinephrine could have more effects on hyperglycemia than norepinephrine based on the investigation of infusion epinephrine and norepinephrine into healthy humans (39). However, there was no clinical study on the differences in the mechanisms between excess of epinephrine and that of norepinephrine on glucose intolerance in patients with phaeochromocytoma.

A recent study elucidated the differences in the actions between epinephrine and norepinephrine on glucose intolerance in patients with phaeochromocytoma (40). The study investigated the association between the changes of urinary metanephrine/normetanephrine (metabolic product of epinephrine/norepinephrine) and those of HOMA-β/HOMA-IR from pre-operation to post-operation in 12 patients with phaeochromocytoma. The study revealed that phaeochromocytoma could lead to glucose intolerance by both impaired insulin secretion and increased insulin resistance. Furthermore, the study demonstrated there could be the differences in the actions on glucose intolerance between excess of epinephrine and that of norepinephrine. The results indicated an excess of epinephrine could affect glucose intolerance mainly by impaired secretion of insulin and that of norepinephrine could affect glucose intolerance mainly by increased insulin resistance. Regarding impaired insulin secretion, the results could be explained by the molecular mechanisms which epinephrine has a higher affinity to adrenergic α2 receptors than norepinephrine. Considering increased insulin resistance, norepinephrine could have stronger effects than epinephrine on increased fatty acids due to lipolysis in adipocytes, which were through adrenergic β3 and α1 receptors. Seeing the contrary affinities to adrenergic α1 and β2 receptors between epinephrine and norepinephrine on glucagon secretion as well as glucose uptake in skeletal muscle together, the results of the clinical study about increased insulin resistance could be consistent with the molecular mechanisms (**Figure 1**).

Hence, the study of different types of the excess of catecholamine should be important for understanding the mechanism of glucose intolerance in patients with phaeochromocytoma. It is also possible that types of the excess of catecholamine affects GLP-1 secretion on phaeochromocytoma. Regarding the association between GLP-1 secretion and excess of catecholamine, there were two previous *in vivo* and *in vitro* studies (41, 42). The *in vitro* study indicated epinephrine could increase GLP-1 secretion through adrenergic α1, α2, and β1 receptors, whereas the *in vivo* study indicated norepinephrine could inhibit GLP-1 secretion through adrenergic α1 and/or α2 receptors (41, 42) (**Table 1**, **Figure 1**). These studies indicated differences in effects on GLP-1 secretion between epinephrine and norepinephrine could exist. Petrák and co-workers did not investigate the different effects on GLP-1 secretion between excess of epinephrine and that of norepinephrine (33). Hence, future studies about the association between GLP-1 and the type of excess of catecholamine might reveal further knowledge of GLP-1 secretion on phaeochromocytoma. Furthermore, they might lead to clarification of the mechanism of increased glucagon secretion after extirpation of phaeochromocytoma, which was seen in the study by Petrák and co-workers.

## Glucose Intolerance On Paraganglioma

Paragangliomas is extra-adrenal non-epithelial tumors originating from neural crest-derived paraganglion cells located in the region of autonomic nervous system ganglia and accompanying nerves with histology like phaeochromocytoma (3). Regarding glucose intolerance in paraganglioma, Elenkova and co-workers noted that 44% (8/18) of patients with paraganglioma had glucose intolerance (11). However, there were no other studies investigating glucose intolerance focused on paraganglioma including differences between glucose intolerance on phaeochromocytoma and that on paraganglioma. The mechanism of glucose intolerance on paraganglioma had never been investigated in clinical studies in the English literature. di Paolo and co-workers previously reported the case of extra-adrenal phaeochromocytoma (paraganglioma) with glucose intolerance due to increased insulin resistance (43). The case had also high norepinephrine secretion. Commonly, paraganglioma produces more norepinephrine than epinephrine (44). Considering the different actions on glucose intolerance between epinephrine and norepinephrine, glucose intolerance in patients with paraganglioma could be caused by increased insulin resistance mainly as the reported case.

## Conclusion

In this article, current perspectives about glucose intolerance in patients with phaeochromocytoma were presented along underlying the mechanism of glucose intolerance. The findings indicated that patients with phaeochromocytoma could have both impaired insulin secretion and increased insulin resistance by an excess of catecholamine. However, the definite mechanism remains unknown, and it has been controversial whether the main factor of glucose intolerance is impaired insulin secretion or increased insulin resistance. Recent advances from the viewpoint of the difference of actions between excess of epinephrine and that of norepinephrine could clarify this controversy. A larger cohort of patients is required to investigate the mechanism and effects of hyperglycemia in pheochromocytoma and paraganglioma.

## Author Contributions

IA wrote the first draft of the manuscript. FI and AKL revised and edited the manuscript. All authors contributed to the article and approved the submitted version.

## Funding

Funding from private practice funding account in Griffith University.

## Conflict of Interest

The authors declare that the research was conducted in the absence of any commercial or financial relationships that could be construed as a potential conflict of interest.
